# MicroRNAs in the Regulation of MMPs and Metastasis

**DOI:** 10.3390/cancers6020625

**Published:** 2014-03-25

**Authors:** Mohammed Abba, Nitin Patil, Heike Allgayer

**Affiliations:** 1 Molecular Oncology of Solid Tumors, German Cancer Research Center (DKFZ), 69120 Heidelberg, Germany; E-Mails: m.abba@dkfz.de (M.A.); n.patil@dkfz.de (N.P.); 2 Department of Experimental Surgery, Medical Faculty Mannheim, Ruprecht Karls University of Heidelberg, Theodor Kutzer Ufer 1-3, 68135 Mannheim, Germany

**Keywords:** microRNAs, MMPs, cancer, metastasis

## Abstract

MicroRNAs are integral molecules in the regulation of numerous physiological cellular processes including cellular differentiation, proliferation, metabolism and apoptosis. Their function transcends normal physiology and extends into several pathological entities including cancer. The matrix metalloproteinases play pivotal roles, not only in tissue remodeling, but also in several physiological and pathological processes, including those supporting cancer progression. Additionally, the contribution of active MMPs in metastatic spread and the establishment of secondary metastasis, via the targeting of several substrates, are also well established. This review focuses on the important miRNAs that have been found to impact cancer progression and metastasis through direct and indirect interactions with the matrix metalloproteinases.

## 1. Introduction

**Figure 1:**
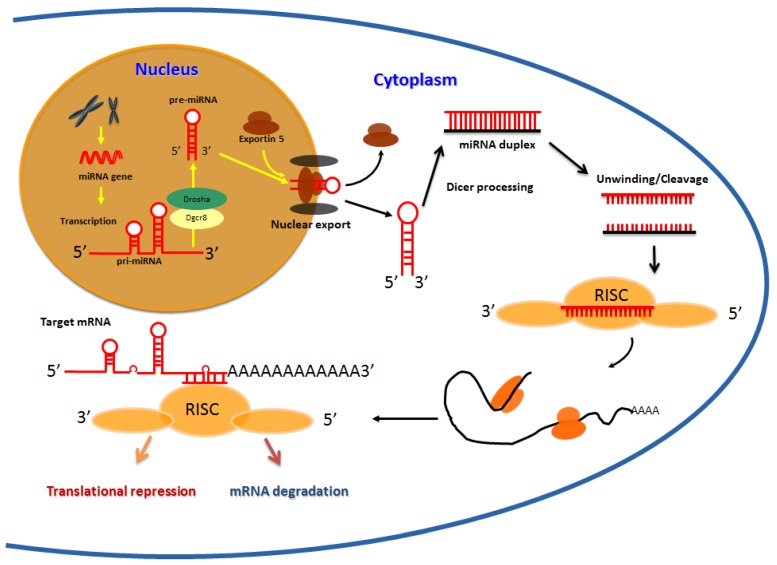
MicroRNA biogenesis: Schematic representation of the events during miRNA biogenesis. The miRNA genes often located in intergenic region are transcribed by RNA polymerase II into local hairpin structures called primary microRNAs (pri-miRNAs) which are then processed to pre-miRNAs by Drosha and Pasha in the cell nucleus. After exportationin the cytoplasm, a ~22 nt duplex is formed due to Dicer processing in association with other cofactors. Mature miRNAs recognize seed sequences in the 3' UTRs of target mRNAs and repress gene expression by either destabilizing target mRNAs or repressing their translation.

MiRNAs encompass a large family of non-coding small RNAs which occur as single-stranded RNAs of ~22 nucleotides (nt) in length (range 19–25 nt) [1,2]. The genes coding for these microRNAs are mostly located within intergenic regions, or within introns of annotated genes, and occur individually or within clusters containing other microRNAs [3,4]. Their genes are transcribed by RNA polymerase II into local hairpin structures called primary microRNAs (pri-miRNAs) and these pri-miRNAs are then processed to pre-miRNAs in the nucleus by the RNAse III enzyme, Drosha, and the double stranded RNA-binding protein, Pasha [5]. Pre-miRNAs are then exported to the cytoplasm by the nuclear export factor Exportin 5 and the Ran-GTP cofactor [6], where they are cleaved by another RNase III type enzyme, Dicer, to generate a ~22 nt RNA duplex (Figure 1). One strand of the miRNA duplex is usually selected as a mature miRNA, and is assembled into an RNA induced silencing complex (RISC), while the other strand is degraded [7]. The RISC complex interacts with the Argonuate proteins and they collectively act to silence target mRNAs [8,9]. The mechanism of mRNA silencing is dependent on the degree of complementarity. In the case of completely aligned miRNA/mRNA pairs, degradation occurs as a consequence of endonucleolytic cleavage resulting from the proteins bound to RISC [9,10,11]. However, in the case of most animals, perfect complementarity rarely exists [12,13,14], and as such the target mRNA cannot be degraded by this mechanism [15]. Consequently, these imperfect miRNA/mRNA pairs are either translationally repressed or silenced independent of the above mentioned mechanism [16,17,18]. The complementarity to the messenger RNA within positions 1–8 of the microRNA is the most crucial parameter for regulation [18], and binding sites on the mRNA are located in most instances on the 3' untranslated region (UTR).

Matrix metalloproteinases (MMPs) are zinc dependent proteases, historically classified, on the basis of their activity, into collagenases, gelatinases, stromelysins and matrilysins (reviewed in [19,20]). These proteases are generally secreted as pro-enzymes (zymogens) and are activated by proteolytic removal of the pro-peptide pro-domain [21]. The consequence of MMP induced proteolysis are several, and include the conversion of structural matrix proteins to signalling molecules, structural changes to matrix proteins and tissue architecture, activation of latent signalling molecules, and alteration of the range of action of signalling molecules. Others include chemo-attraction, proliferation, differentiation and cell survival [21,22,23,24]. From recent data out of several publications, it is evident that the relationship between MMP expression and disease it not quite simple, as increased expression of MMPs does not always tally with disease progression or severity, moreover, we do not have 23 MMPs just to promote disease.

The activity of MMPs is tightly regulated and even though many proteases can be found solubilised in plasma, they are kept in check either by natural MMP inhibitors, or are simply inactive as pro-enzymes [25,26]. Tissue inhibitors of metalloproteinases (TIMPs) are the major cellular inhibitors of the MMPs, but interestingly, elevated levels of the tissue inhibitor TIMP-1 is found in several cancer types and elevated levels of TIMP-1 and -2 have been correlated with poor prognosis and cancer progression [27]. The relationship between MMPs and the TIMPs is not always consistent with reports showing increased tumorigenesis and aggressiveness as well as inhibition of tumor growth with elevated levels of TIMPs [28,29]. Such studies of MMPs and their natural inhibitors with no direct correlation raises many questions on how pro-MMPs get catalytically activated in the presence of elevated levels of TIMP, as observed in tumors, and whether elevated levels of MMPs denote increased functional activity, or simply reflect their de-regulation alongside their natural TIMPs [30].

It is nonetheless pertinent to point out that the crucial role of MMPs in cancer is mediated largely by the extracellular matrix (ECM) degradation which helps in cell invasion and metastasis [20,21]. A strong correlation between the altered expressions of MMPs at RNA or protein level in various cancer types is associated with poor disease prognosis. With only two exceptions from two independent studies, the presence or elevated levels of MMP-1, -2, -3, -7, -9, -13 and -14 in primary tumors or in metastases have generally been associated with poor tumor differentiation, invasion stage of cancer, shorter survival time and metastasis to secondary organs (reviewed in [30]). The first exception is with MMP-8 where in breast cancer, reduced expression was associated with enhanced metastasis of tumor cells and in null mice, increased susceptibility to skin tumor development was observed with reduced MMP-8 expression [31,32]. The second instance arose when MMP-3 knockout mice showed enhanced skin tumor development [33].

## 2. MicroRNAs, Cancer and Metastasis

Many miRNAs have been linked to cancer [34,35,36,37]. Expression analysis studies have demonstrated aberrant miRNA expression in tumors compared to normal tissues and miRNAs are deregulated in an array of solid cancers as well as haematological malignancies [34,38]. The findings that miRNAs have a role in cancer is supported by the fact that about 50% of miRNA genes are located in cancer associated genomic regions, or in fragile sites [38], further strengthening the evidence that miRNAs do play a crucial role in cancer. As a result, human miRNAs are likely to be highly useful as biomarkers, especially for future cancer diagnostics, and are emerging as attractive targets for disease intervention. The roll call for cancer associated miRNAs has grown very rapidly and, at present over 1800 precursor loops and 2500 mature microRNAs are recognized [39].

Deregulated miRNA expression can influence a broad range of biological processes that are involved in tumorigenesis such as transcription, signal transduction, cellular proliferation, differentiation, apoptosis and epithelial to mesenchymal transition (EMT) [40,41,42,43]. Interestingly, the aberrant regulation of certain miRNAs has been shown to cut across several cancer types, for instance miR-21 is commonly up-regulated in breast, colon, lung, pancreas, prostate, stomach, cervical, ovarian, hepato-billiary and head and neck cancers as well as in B-cell lymphoma and chronic lymphocytic leukaemia [44,45,46,47,48], where different tumor suppressor mRNAs inclusive of programmed cell death 4 (Pdcd4), phosphatase and tensin homolog (PTEN) and tropomyosin 1 (alpha) (TPM1), to mention a few, are targeted. Further examples include miR-155 and miR-34 [49]. In some instances, the deregulated expression of a miR, including implicated mechanisms, has been established to be a useful prognostic marker [50,51,52,53,54].

The detection of circulating miRs in blood and serum, and their altered expression levels in diseased states has opened up new opportunities in cancer diagnosis. A number of publications have exploited this possibility. Notable examples include the implication of miR-21 in the survival of B-cell lymphoma patients [55] and the serum expression of miR-141 in differentiating healthy individuals and those with prostate cancer [56]. Furthermore, in ovarian cancer, the strong correlation of miRs-21, -141, -200a, -200c, -200b, -203, -205 and -214 in tissues and tumor exosomes prompted the authors to conclude that these microRNAs could serve as a surrogate signature in this cancer type. A similar conclusion was also made in lung adenocarcinomas with a small cohort of 27 patients [57,58]. Likewise, Keller *et al.*, using a microarray platform, were able to discern and validate 24 microRNAs from blood cells which distinguished patients with non small cell lung cancer (NSCLC) from normal controls [59].

## 3. MicroRNAs and MMPs in Cancer Progression and Metastasis

A growing number of miRNAs have been implicated in the regulation of the MMPs culminating in a disruption of distinct MMP functions and since these functions are ingrained in several of the processes that support cancer progression such as ECM remodelling, epithelial to mesenchymal transition (EMT) and angiogenesis, these contributions could have significant consequences. It is pertinent to mention at this point that several microRNAs have been implicated in different pathological processes however, only those related to cancer progression and metastasis are discussed here and elaborated in Table 1. Furthermore, since all the MMPs have not been explored to the same intensity, especially in the context of MMP/miRNA interactions as it relates to cancer progression and metastasis, this review is skewed in favour of those MMPs where the literature is more abundant.

## 4. Matrix Metallopeptidase 1 (MMP-1)

This MMP belongs to the collagenase family of the metalloproteinases and is specifically responsible for the breakdown of collagens I to III [60]. A few reports have evaluated the contribution of MMP-1/miRNA interactions in tumorigenesis. In a microarray study on paired oral tongue squamous cell carcinoma (OTSCC) cell lines with different metastatic potential, Liu and colleagues identified miR-222 as a definitive regulator of MMP1 expression, mediated by both direct cis- (MMP-1) and an indirect trans-regulatory mechanisms targeting superoxide dismutase 2 (SOD2), thereby inhibiting cell invasion and potentially metastasis [61].

**Table 1:** Summary of microRNA/MMP linked interactions in cancer: Individual microRNAs and targeting MMPs directly or indirectly via other proteins leading up to cancer progression and metastasis in different cancer entities are outlined. Also included the possible pathways implicated in the mediation of observed phenotype.

Sr.No	microRNA	MMP Type and target molecule	Cancer type	Phenotype investigated	Pathway	Reference
1	let-7	MMP-9	Melanoma	Cell proliferation and migration	-	[62]
2	let-7	MMP-14, ERK1/2 activation	Pancreatic ductal adenocarcinoma	NA	ERK1/2 activation, TGF-β1 signaling	[63]
3	let-7	Focal adhesion kinase (FAK), AKT, ERK, MMP-2 and MMP-9	Glioblastoma	Migration and invasion	AKT and ERK	[64]
4	miR-9	MMP-2, MMP-9 and VEGFA	Uveal melanoma	Migration and invasion	NF-κB1 signaling	[65]
5	miR-9	MMP-14	Neuroblastoma	Invasion, metastasis, and angiogenesis	-	[66]
6	miR-10b	MMP-9, E-cadherin and vimentin	Nasopharyngeal carcinoma cells	Proliferation, migration, invasion	-	[67]
7	miR-10b	MMP-14 and uPAR	Glioma	Cell invasiveness	-	[68]
8	miR-10b	MMP-2, EGFR.	Glioblastoma multiforme	Apoptosis invasion and migration	EGFR pathways	[69]
9	miR-15b	MMP-3	Glioma	Cell invasiveness	MEK-ERK pathway	[70]
10	miR-17	MMP-3	Hepatocellular carcinoma	Migration and invasion	p-AKT	[71]
11	miR-21	RECK, MMP-9	Prostate cancer	NA	-	[72]
12	miR-21	Phospho-c-Jun, MMP-2, MMP-9	Hepatocellular carcinoma	Migration and invasion	-	[73]
13	miR-21	RECK, MMP-2	Glioma	Apoptosis, migration, and invasiveness	-	[74]
14	miR-21	MMP-2, EGFR.	Glioblastoma multiforme	Apoptosis invasion and migration	EGFR pathways	[69]
15	miR-26a	MMP-2	Lung cancer	Migration, invasion and metastasis	AKT phosphorylation	[75]
16	miR-29b	MMP-2	Colon cancer	Migration	-	[76]
17	miR-29b	MMP-2	Hepatocellular carcinoma	Tumor angiogenesis, invasion, and metastasis	VEGFR-2-signaling	[77]
18	miR-29b	MMP-2, Mcl-1, COL1A1, and COL4A1	Prostate cancer	invasion and metastasis	-	[78]
19	miR-29c	MMP-2	Nerve sheath tumours	Cell invasion and migration	-	[79]
20	miR-30d	SOCS1, phospho-STAT3, MMP-2 and MMP-9	Prostate cancer	Proliferation and invasion	STAT3 signalling	[80]
21	miR-34a	Fra-1, p53 MMP-1 and MMP-9	Colon cancer	Migration and invasion	-	[81]
22	miR-92a	MMP-2 and -9	Lung cancer	Migration and invasion	STAT3 signaling	[82]
23	miR-101	Enhancer of zeste homolog 2 (EZH2), CDH1 and MMP-2	Lung cancer	Cell invasiveness	-	[83]
24	miR-106b	MMP-2	Breast cancer	Migration and invasion	ERK signaling cascade	[84]
25	miR-125b	MMP-2 and MMP-9	Glioblastoma	Invasion	-	[85]
26	miR‑133	MMP‑14	Lung cancer	Cell proliferation, migration and invasion	-	[86]
27	miR-138	RhoC, MMP-2 and MMP-9	Cholangiocarcinoma	Proliferation, migration and invasion	p-ERK signaling	[87]
28	miR-139	IGF-IR and MMP-2	Colorectal cancer	Migration, invasion and metastasis	IGF-IR/MEK/ERK signaling	[88]
29	miR-143	MMP-13	Prostate cancer	Migration and invasion	-	[89]
30	miR-143	MMP-2 and MMP-9	Pancreatic cancer	Migration and invasion	-	[90]
31	miR-143	MMP-13	Osteosarcoma	Cell invasiveness	-	[91]
32	miR-145	Ets1, MMP-1 and -9	Gastric cancer	Invasion, metastasis, and angiogenesis	-	[92]
33	miR-146a	MMP-1, uPA, and uPAR	Brain cancer	Migration, invasion and metastasis	-	[93]
34	miR-146a	MMP-16	Colon cancer	Invasion	-	[94]
35	miR-149	MMP-2 and CyclinD1	Glioma	Proliferation and invasion	AKT signaling	[95]
36	miR-152	MMP-3	Glioma	Cell invasiveness	MEK-ERK pathway	[70]
37	miR-181b	MMP-2 and MMP-9	Hepatocellular carcinomas	Migration and invasion	TGF- β, Smad signaling	[96]
38	miR-182	MMP-9, RECK	Breast cancer	cell invasion and colony formation ability	-	[97]
39	miR-196b	Vimentin, MMP-2 and MMP-9	Gastric cancer	Migration and invasion	-	[98]
40	miR-203	MMP-9 and Robo1	Glioblastoma	Proliferation, migration, and invasion	ERK phosphorylation	[99]
41	miR-206	MMP-2 and MMP-9	Breast cancer	Invasion and migration	-	[100]
42	miR-211	MMP-9	Glioblastoma multiforme	Cell invasion and migration	-	[101]
43	miR-218	LEF1, MMP-2, -7 and -9	Glioblastoma multiforme	Invasion	-	[102]
44	miR-218	MMP-9	Gliomas	Cell invasiveness	IKK-β/NF-κB pathway	[103]
45	miR-224	MMP-9 via targeting HOXD10	Human hepatocellular carcinoma	Migration and invasion	-	[104]
46	miR-338-3p	SMO and MMP-9	Hepatocellular carcinoma	Invasion and metastasis	-	[105]
47	miR-340	MMP-2 and MMP-9	Breast cancer	Tumor cell growth, migration, and invasion	-	[106]
48	miR-430	ERK, MMP-2 and MMP-9	Bladder cancer	Proliferation, migration and colony formation ablility	-	[107]
49	miR-451	Akt1, CyclinD1, MMP-2, MMP-9 and Bcl-2	Glioblastoma	Proliferation, invasion and apoptosis	PI3K/AKT signaling	[108]
50	miR-491	MMP-9	Hepatocellular carcinoma	Migration	-	[109]
51	miR-491-5p	MMP-9	Glioblastoma multiforme	Invasion	-	[110]
52	miRNA-590-3p	PI3K, Akt, MMP-2 and MMP-9	Bladder cancer	Proliferation, migration and colony-formation	PI3K, Akt signaling	[111]
53	miR-874	MMP-2 and -9, Aquaporin-3	Human gastric cancer	Cell migration and invasion assays and *in vivo* tumorigenicity	-	[112]
54	miR-874	MMP-2 and uPA	Non-small cell lung cancer	Tumor cell invasiveness and *in vivo* tumor growth	-	[113]
55	miR-885-5p	MMP-9	Glioblastoma multiforme	Invasion	-	[110]

In gastric cancer, the v-ets erythroblastosis virus E26 oncogene homolog 1 (Ets1) had been implicated in tumor development and progression, in part by trans-activating MMPs-1 and -9. MiR-145 was found to directly target the 3'-UTR of Ets1-mRNA and overexpression or knockdown of this miRNA altered both the mRNA and protein levels of Ets1 and those of MMPs-1 and -9 with subsequent inhibition of invasion, metastasis, and angiogenesis of gastric cancer cells [92].

Moreover, in investigating brain specific metastasis, Hwang and colleagues discovered that miR-146a was significantly suppressed in the brain-trophic metastatic LvBr2 breast cancer cells in comparison to the parental cell line, and that this microRNA lowered the expression of MMP-1 and the serine protease plasminogen activator, urokinase (uPA) and its receptor. This was, however, an indirect effect purported to be mediated via the heterogeneous nuclear ribonucleoprotein C1/C2 (hnRNPC) [93].

## 5. Matrix Metallopeptidase 2 (MMP-2)

MMP-2, also known as the 72 kDa form of type IV collagenase has, in addition to tissue remodelling been intricately linked to embryonic development [60]. Torg-Winchester, multicentricosteolysis and arthritis syndromes have all been associated with mutations in this gene [114].

In breast cancer, the transmembrane heparan sulfate proteoglycan (syndecan-1) has been linked to poor outcomes and MMP-2 was found amongst several other genes to be overexpressed in syndecan-1 deficient cells, with miR-10b to proven contribute to this effect by directly interacting with the 3' UTR of syndecan-1 [115]. In an interesting study looking at bone specific metastasis in breast cancer, MMP-2 was found to be significantly overexpressed in bone as opposed to orthotopically located breast cells. Consequently, miR-106b was found to be not only substantially decreased at metastatic sites, but also to directly target MMP-2. The loss of miR-106b therefore accounted for increased MMP-2 expression, leading to enhanced migration and invasion of breast cancer cells [84].

In prostate cancer, miR-29b was identified to be upregulated in response to c-myc promoter binding protein (MBP-1), and as a result, the expression of a number of oncogenic proteins including MMP-2, myeloid cell leukemia sequence 1 (Mcl-1), collagen, type I, alpha 1 (COL1A1), and collagen, type IV, alpha 1 (COL4A1) were all down regulated and MMP-2 was experimentally confirmed to be a direct target of miR-29b [78]. In a comparison between castration-resistant- (CRPC) and androgen-dependent prostate cancer (ADPC) tissues, the expression of miR-146a was found to be significantly decreased in the castration resistant tissues and thus postulated to be a potential molecule mediating androgen sensitivity. MiR-146a directly repressed epidermal growth factor receptor (EGFR) and consequently MMP-2, *in vitro* cell growth, colony formation, and migration as well as *in vivo* tumorigenicity and angiogenesis were all reduced [116]. A fractional extract obtained from American ginseng plant has been demonstrated to have anti-cancer properties in colorectal cancer and its mechanism of drug action was discovered to be due to the overexpression of miR-29b, which regulates MMP-2 expression [76] as was observed in prostate cancer. Furthermore in a tumor-normal comparison of colorectal cancer (CRC) tissues, miR 139 was found to be down regulated in the cancer as compared to normal tissues and the re-expression of this miRNA led to the suppression of CRC cell metastasis and invasion *in vitro* and *in vivo*. Type I insulin-like growth factor receptor (IGF-IR) was identified as a direct target of miR-139 in this instance, but a decrease in the cellular protein expression of MMP-2, as well as activity in the supernatant of two cell lines was observed as a direct consequence [88].

In lung cancer, miR-26a expression levels were observed to be higher in stages with nodal metastasis than in primary cancer and leads to increased lung cancer aggression. The direct target in this instance was PTEN and MMP-2 was indirectly enhanced [75]. Similarly, miR-874 was identified using *in-situ* hybridization as drastically suppressed in NSCLC tissues as compared to normal samples, and MMP-2 was predicted and confirmed to be its most significant putative target with 3 binding sites in its 3' UTR. Enhanced expression of miR-874 led to disappearance of cancer stem phenotype in a CD133-positive population that was associated with reduced MMP-2 and uPA protein levels [113].

The role of miR-149 was explored in glioblastoma where it was found to inhibit the expression of MMP-2, p-AKT1, proliferating cell nuclear antigen (PCNA), cyclin D1, as well as proliferation and invasion in U251 cells [95]. Also, miRs-21 and -10b were show to be significantly elevated in glioblastoma multiforme (GBM), and the experimental inhibition of these miRs in glioblastoma cells led to a significant decrease in MMP-2, EGFR, RhoC expression, and a concomitant increase in the tumor suppressors Pdcd4, tropomyosin (TPMI) and HoxD. These modulations were more significant when miR-21 and -10b were concurrently inhibited as compared to individual silencing [69].

A low expression of miR-29b, often associated with poor-recurrence free survival in hepatocellular cancer (HCC), was experimentally found to be a direct target of MMP-2 with significant effects on angiogenesis and invasion. Tumors derived from miR-29b-expressing HCC cells demonstrated a significant reduction in microvessel density and in intra-hepatic metastatic capacity compared with those from the control group in mice [77]. In deciphering the microRNAs that drove malignant transformation of neurofibromas to malignant peripheral nerve sheath tumours (MPNST), miR-29c was identified as one of those that were significantly down-regulated and MMP-2 together with a host of other genes involved in cell migration and invasion to be direct targets. Functional studies in a MPNST cell line, sNF96.2, using a mimic of the mature miR-29c showed reduced invasion, whereas no change in proliferation was seen [79]. In addition to the microRNAs that impact MMP-2 as elucidated above, a number of miRs concomitantly deregulate MMPs-2 and -9, and these are elaborated in the MMP-9 section.

## 6. Matrix Metallopeptidase 3 (MMP-3)

MMP-3, also known as stromelysin-1, is capable of activating MMPs -1, -7 and -9 and plays a significant role in connective tissue remodelling [60,117]. Two reports have implicated microRNAs in the regulation of MMP-3. In an attempt to analyse the regulation of T3/TR-mediated tumor migration in HCC, Lin and colleagues identified miR-17 as being a transcriptionally down-regulated target of TR whose modulation was paralleled by enhanced p-AKT expression. The overexpression of miR-17 markedly inhibited cell migration and invasion via suppression of MMP-3 [71]. In an attempt to define a signature that defines tumor aggression in gliomas, Zeng and colleagues, profiled miRNAs and also the mRNAs that could be targeted by those signature molecules, and identified MMP-3 as a strong invasion associated molecule that was targeted by miR-152. Moreover, a preliminary pathway study indicated that miR-152 together with miR-15b deactivated the mitogen-activated protein kinases (MEK-ERK) pathway [70].

## 7. Matrix Metallopeptidase 9 (MMP-9)

In addition to its normal physiological functions, MMP-9 (92 kDa collagenase IV) is involved in the development of several human malignancies and facilitates tumor progression, invasion, metastasis and angiogenesis [60,118]. The activation of all MMPs in general is a tightly regulated process, consisting of tissue inhibitors and the need for activation from the pro-inactive form into an active form. In the case of MMP-9 this activation occurs principally via MMP-2 and indirectly via an activation axis made up of TIMP-2 and MT1-MMP. A number of publications have also implicated a common trans-activation mechanism for both MMPs such as by noradrenaline in hypothalamic supra-optic nuclei, and the ERK1/2 pathways [119,120].

In bladder cancer, the mitochondrial transcription factor a (TFAM), is commonly overexpressed, and this overexpression frequently coincides with the down regulation of miR-590-3p which was identified to directly target TFAM. As a consequence, the expression of MMP-2 and -9, including phosphatidylinositide 3 (PI3) kinase, were all down regulated, thus suggesting this microRNA to be an indirect regulator of MMPs-2 and -9 [111]. Also down regulated in bladder cancer is miR-430, whose overexpression also inhibits cell proliferation, migration and colony formation that is mediated in part via MMP-2 and -9 as indirect targets [107].

Wu and colleagues discovered that endogenous miR-340 expression was down regulated in aggressive breast cancer cell lines as well as patients tissues. They demonstrated that miR-340 suppresses tumor migration and invasion by directly targeting c-Met and consequently MMP-2 and -9 [106]. Additionally, miR-206 was identified as tumor suppressor in breast cancer cell lines and was found to mediate this effect by directly targeting cell division cycle 42 (CDC42), resulting in down regulation of its protein expression together with that of MMP-2 and MMP-9 [100]. MiR-182 identified as transcriptional target of beta-catenin interacts and suppresses the expression of the MMP- inhibitor reversion-inducing cysteine-rich protein with kazal motif (RECK) in breast cancer cell lines, and as a result of this inhibition, the activity of MMP-9 and consequently cell invasion and colony formation were affected. An inverse association between miR-182 and RECK was demonstrated in breast cancer tissues [97]. The phenomena of RECK down modulation by microRNAs was also observed in prostate cancer cell lines with miR-21 [72].

Similarly in gastric cancer, the water transporting protein aquaporin-3 (AQP3) is an important oncogenic protein which was experimentally validated to be targeted by miR-874. Ectopic expression of miR-874 suppressed tumor migration and invasion and down regulated MMP-2, -9 and -14 [112]. Also in gastric cancer, the tumor suppressor E26 transformation-specific sequence (ETS)-2 regulates miR-196b expression and investigations in clinical specimens revealed an inverse correlation between the two. Interestingly, the expression of MMP-2, MMP-9 and Vimentin were enhanced in miR-196b transfected cells and upon knockdown of (ETS)-2 protein. The loss of (ETS)-2 protein or overexpression of miR-196b leads to an increase in migration and invasion of gastric cancer cell lines [98].

In cholangiocarcinoma, Wang and colleagues identified miR-138 as a significantly down regulated molecule using real time PCR, and equally identified RhoC as a direct target of this microRNA. The upregulation of RhoC that occurs as a result of down regulation of miR-138 promoted malignant progression involving the increased expression of p-ERK, MMP-2 and MMP-9, also via an indirect mechanism [87].

Using a miRNA microarray comparison of prostate cancer and normal cell lines, miR-30d was found to be overexpressed in the cancer cell lines, and experimentally enhanced proliferation and invasion *in vitro*. This microRNA was found to directly suppress cytokine signalling 1 (SOCS1) and thereby down modulate the expression of signal transducer and activator of transcription 3 (p-STAT3), MMP-2 and MMP-9 [80].

In pancreatic cancer, the overexpression of miR-143 significantly decreased the protein levels of MMP-2 and MMP-9, lowered the constitutive activities of RhoA, Rac1, and CDC42 GTPases, and also significantly inhibited cell migration and invasion of tumor cells *in vivo* and *in vitro*. In this study, GEF1, GEF2 and K-Ras were found to be direct targets of miR-143 [90].

The NF-κB pathway is often activated in uveal melanoma and miR-9 was demonstrated to directly modulate NF-κB expression and also that of its downstream targets MMP-2, MMP-9 and vascular endothelial growth factor (VEGF)-a, which are all down regulated upon overexpression of miR-9 [65]. In mouse melanoma cells, Let-7b was identified to target the extracellular matrix metalloproteinase inducer (EMMPRIN). Suppressing its expression also indirectly suppressed MMP-9, and consequently, cellular proliferation, proliferation and metastasis are all reduced [62].

In investigating the role of miR-26a in lung cancer metastasis, Liu and colleagues demonstrated that miR-26a significantly enhanced migration and invasion as well as up regulated the expression of MMP-2, VEGF, Twist and beta catenin [75].

In a diet induced mouse model of HCC, miR-181b and miR-181d were found to be upregulated, and the levels of tissue inhibitor of metalloproteinase 3 (TIMP3), was suppressed as a result of direct interaction of these microRNAs. This also enhanced the activity of MMP-2 and MMP-9, promoting migration and invasion that was reversible by modulating TIMP3 levels [96]. Equally, the tumor suppressor, PDCD4 is directly targeted by miR-21 in HCC and as a result, the downstream molecular signalling cascade including MMP-2, MMP-9 and phospho c-jun were all shown to be affected. MiR-21 is associated with enhanced tumor invasion and migration in several malignancies [44,73]. In an attempt to differentiate poor and well differentiated HCC tissues, miR-491 was identified as the most significantly down regulated miRNA in poorly differentiated HCC. *In vitro* experiments showed that ectopic expression of miR-491 resulted in decreased expression of MMP-2 and MMP-9 and suppressed migratory capacity [109]. Following the identification of miR-338-3p as down regulated in aggressive hepatocellular carcinoma, Huang *et al.* decided to explore the mechanism and identified smoothened (SMO) as a direct target of this microRNA. The expression of MMP-9 was also indirectly affected whereby inhibition of miR-338-3p was found to up regulate SMO and MMP-9 expression in HCC cells [105].

MiR-451 was found to directly impact glioblastoma cell proliferation, invasion, and apoptosis by down regulating the expression of Akt1, CyclinD1, MMP-2, MMP-9 and B-Cell CLL/lymphoma 2 (Bcl-2), however, no direct interaction of any of these mRNAs were found. Instead, the Phosphoinositide-3-kinase (PI3K)/AKT signalling pathway was implicated [108]. Likewise, the expression of miR-218 is low in glioblastoma tissues and this correlated inversely with the expression of mRNAs of MMPs-2, -7 and -9. The Lymphoid enhancer-binding factor-1 (LEF1) was identified to be a direct target of miR-218 with consequent effects on LEF1 and MMP-9 protein levels, indicating that miR-218 directly targets LEF1 resulting in reduced synthesis of MMP-9 [102]. Yet again, miR-203 was identified as potential tumor suppressor in glioblastoma since it is located at a commonly deleted region of chromosome 14q. This was validated in a larger patient cohort and roundabout homolog 1 (Robo1) was identified as a relevant direct target. In addition, miR-203 expression was found to suppress ERK phosphorylation and MMP-9 as a direct consequence [99]. In a study specifically seeking to identify microRNAs regulating MMP-9, fourteen positive and thirty one negatively correlating microRNAs were identified of which miR-491-5p was found to not only directly target MMP-9, but also supress glioma cell invasion [110]. Moreover, in glioma cells, miR-218 was shown to directly target the NF-kB suppressor IKK/Beta, thereby activating the NF-kB pathway and MMP-9 [103].

## 8. MMPs-11, -13, -14 and -16

Let-7c directly destabilizes MMP-11 and pre-B-cell leukemia homeobox 3 **(**PBX3) mRNA and was found to modulate tumor metastasis in colorectal cancer [121].

MMP-13 was found to be directly targeted by miR-125b, leading to the suppression of cell migration and invasion in bladder cancer cell lines [122]. Similarly, miR-143 overexpression led to the inhibition of migration and invasion in prostate cancer lines, which was due to direct inhibition of MMP-13 mRNA [89]. In an independent investigation, miR-143 was found to be down regulated in a lung cancer specific mouse metastatic model, and overexpression of miR-143 was associated with decreased invasiveness but not proliferation [91].

In pancreatic ductal adenocarcinoma (PDAC), let-7 indirectly regulates the expression of MMP-14 and ERK1/2 activation [63]. Xu and Wang demonstrated that overexpression of miR-133a in lung cancer lines resulted in reduced cellular proliferation, migration and invasion as a result of targeting MMP-14 [86]. In neuroblastoma tissues, MMP-14 expression was monitored using immunostaining and correlation to clinical data revealed an association of MMP-14 expression with poor patient survival. Moreover, miR-9 was found to inhibit MMP-14 expression by directly interacting with the 3' UTR of MMP-14, and was responsible for a loss of invasion, metastasis and angiogenesis [66]. In glioma cells, MMP-14 and uPAR expression were found to be modulated by miR-10b [68].

MMP-16 has been demonstrated to play a crucial role in migration and invasion of glioma cells and to be directly targeted by miR-146b [123]. A very similar phenomenon was observed in a colorectal cancer cell line (Caco-2) in which miR-146a directly regulated the expression of MMP-16. Overexpression of miR-146a resulted in decreased cellular motility [94].

## 9. Conclusions

Although the list of miRNAs targeting MMPs and/or their regulators, as directly or indirectly discussed above is by no means exhaustive, a plausible inference, at least from the studies so far published, is that microRNA regulation of MMP expression is an important and culpable mechanism driving cancer progression.

In view of their critical significance in cancer progression, several attempts have been made at targeting MMPs, with mixed success. Many molecules or antibodies developed against MMPs were broad spectrum and non-specific. Many companies are currently exploring other possibilities of anti-MMP therapy in the form of oral or parenteral formulations with minimal side effects in cancer, but have failed, largely due to toxic effects of the drugs. It remains to be seen whether new developments targeting MMPs or rather targeting microRNAs regulating MMPs can be successfully used to delay metastasis or stop cancer progression. Although, our current knowledge of MMPs seems to be every extensive, not all the MMPs have been explored to the same extent and additional research efforts in the basic sciences are definitely required to bridge this gap.
